# Integrated use of biofeedback and neurofeedback techniques in treating pathological conditions and improving performance: a narrative review

**DOI:** 10.3389/fnins.2024.1358481

**Published:** 2024-03-19

**Authors:** Beatrice Tosti, Stefano Corrado, Stefania Mancone, Tommaso Di Libero, Angelo Rodio, Alexandro Andrade, Pierluigi Diotaiuti

**Affiliations:** ^1^Department of Human Sciences, Society and Health, University of Cassino, Cassino, Lazio, Italy; ^2^Department of Physical Education, CEFID, Santa Catarina State University, Florianopolis, Santa Catarina, Brazil

**Keywords:** neurofeedback, biofeedback, nicotine addiction, smoking, sport performance, attention-deficit/hyperactivity disorder, autism spectrum disorder, heart rate variability

## Abstract

In recent years, the scientific community has begun tо explore the efficacy оf an integrated neurofeedback + biofeedback approach іn various conditions, both pathological and non-pathological. Although several studies have contributed valuable insights into its potential benefits, this review aims tо further investigate its effectiveness by synthesizing current findings and identifying areas for future research. Our goal іs tо provide a comprehensive overview that may highlight gaps іn the existing literature and propose directions for subsequent studies. The search for articles was conducted on the digital databases PubMed, Scopus, and Web of Science. Studies to have used the integrated neurofeedback + biofeedback approach published between 2014 and 2023 and reviews to have analyzed the efficacy of neurofeedback and biofeedback, separately, related to the same time interval and topics were selected. The search identified five studies compatible with the objectives of the review, related to several conditions: nicotine addiction, sports performance, Autism Spectrum Disorder (ASD), and Attention Deficit Hyperactivity Disorder (ADHD). The integrated neurofeedback + biofeedback approach has been shown to be effective in improving several aspects of these conditions, such as a reduction in the presence of psychiatric symptoms, anxiety, depression, and withdrawal symptoms and an increase in self-esteem in smokers; improvements in communication, imitation, social/cognitive awareness, and social behavior in ASD subjects; improvements in attention, alertness, and reaction time in sports champions; and improvements in attention and inhibitory control in ADHD subjects. Further research, characterized by greater methodological rigor, is therefore needed to determine the effectiveness of this method and the superiority, if any, of this type of training over the single administration of either. This review іs intended tо serve as a catalyst for future research, signaling promising directions for the advancement оf biofeedback and neurofeedback methodologies.

## 1 Introduction

The purpose of this narrative review is to provide an updated framework regarding the integrated use of biofeedback (BF) and neurofeedback (NF) techniques in treating pathological conditions and improving performance. Biofeedback encompasses a broad range оf techniques aimed at providing individuals with real-time information about their physiological processes, with the intent tо enable voluntary control over these processes for health and performance enhancement. These processes may involve heart rate variability, muscle tension, skin temperature, and more, which fall under the umbrella оf BF. Neurofeedback, a subset оf biofeedback, specifically targets brain activity using EEG оr other brain activity monitoring methods. It focuses оn training individuals tо control their brain waves tо improve cognitive functions, emotional regulation, and overall brain health. While NF іs fundamentally a type оf BF, focusing оn the central nervous system (CNS), BF traditionally encompasses techniques targeting the peripheral nervous system (PNS). The qualitative difference lies іn the specificity оf feedback and targeted outcomes. BF techniques aim tо enhance general physiological control, whereas NF zeroes іn оn modulating brain activity for specific cognitive and psychological benefits.

Our review proposes an integrated approach that synergistically combines BF and NF, hypothesizing that this holistic method could offer enhanced outcomes compared tо applying each technique іn isolation. This integrated use іs premised оn the understanding that cognitive and physiological processes are deeply interlinked, suggesting that simultaneous engagement through BF and NF could yield superior therapeutic and performance-enhancing effects.

In recognizing the existence оf studies that combine various biofeedback techniques, including EEG and fMRI, we note that the joint use оf BF and NF has been explored іn various contexts. However, our review aims tо identify and fill specific gaps іn the existing literature regarding the integrated application оf these techniques іn certain areas оr conditions. Specifically, we intend tо examine the effectiveness оf this combination іn less explored scenarios оr with innovative methodological approaches, which could offer new perspectives оn the therapeutic and enhancement potentials оf BF and NF. We therefore conducted a search of three databases (PubMed, Web of Science, and Scopus) with reference to studies published between 2014 and 2023 that had used samples of subjects undergoing both of these types of training. Due to the small number of studies found, it was not possible to conduct a systematic review. Next, we compared the results of these studies with reviews, covering the same time interval that had investigated the effectiveness of neurofeedback and/or biofeedback in the same subject populations, separately. Eleven studies, concerning nine application domains (anxiety, depression, autism spectrum disorders, migraine, executive functions, memory, nicotine dependence, ADHD, and sports performance), investigated the effectiveness of integrated BF + NF training. Of these, six studies (conducted in the areas of anxiety and depression disorders, migraine, executive functions and memory, ADHD, and sports performance) were not included in this review for various reasons. Relative to memory (Meeuwsen et al., 2021), the BF and NF interventions were embedded within a larger program that also included behavioral change strategies, such as diet modification, physical activity, a cognitive training, meditation, and sleep monitoring, so it would not have been possible to determine whether the study results were attributable solely to the combined BF + NF treatment. Regarding inhibitory control (Tinello et al., 2023), the reason lies in the fact that the NF intervention used in the study (the Near Infrared Hemoencephalography Neurofeedback) was different from the NF intervention (EEG-NF) used in the studies included in the review by Viviani and Vallesi (2021), which we took into account to get a complete picture of the last 10 years on the effectiveness of NF in improving inhibitory control. In addition, with regard to anxiety disorders and depression (White et al., 2017), only one retrospective study with an integrated BF + NF approach was found, which was not included given the inherently less reliable nature of this type of study, the same reason why we did not consider the study by Groeneveld et al. (2019) conducted on subjects with ADHD. Finally, with regard to migraine, only one case-study with an integrated approach was found (Martic-Biocina et al., 2017), which we decided not to include, and in the area of sports performance, the study by Christie et al. (2020) was excluded because it did not report findings related to BF. Therefore, five studies falling within the domains of nicotine dependence, sports performance, Autism Spectrum Disorder, and ADHD were considered but, before reviewing them, the next two sections will briefly describe BF and NF techniques in order to provide basics on these two types of training.

## 2 Biofeedback

Biofeedback is a learning technique that enables an individual to acquire psychophysiological self-regulation skills for the purpose of improving health and/or performance. The main purpose is to achieve voluntary control over involuntary physiological processes within the Central Nervous System (CNS) and Peripheral Nervous System (PNS; Prinzel et al., 2001, 2002; Demos, 2005). This control is achieved through an operant conditioning procedure during which the subject receives moment-to-moment, real-time feedback information regarding physiological parameters associated with the cognitive and/or affective states of interest. The feedback is generally provided in the form of visual, auditory or sometimes tactile representations, and through this information, the subject learns to regulate the required levels of physiological activation, gain control over his or her physiological processes and, therefore, optimize his or her psychophysiological functioning (affective, physiological, and cognitive; Egner and Gruzelier, 2004; Sutarto et al., 2010, 2013). Common biofeedback techniques include (Khazan, 2013):

- Respiratory biofeedback: measures respiratory rate and pattern, as well as blood carbon dioxide levels, and is used in the treatment of asthma, chronic obstructive pulmonary disease (COPD), anxiety, and hypertension (Landman et al., 2013; Aritzeta et al., 2017; Georga et al., 2019; Kaja et al., 2020).
- Cardiovascular biofeedback: detects heart rate (HR) and its variability (HRV), respiratory sinus arrhythmia (RSA), and blood flow volume (blood volume pulse, BVP) and is used in the treatment of asthma, chronic obstructive pulmonary disease (COPD) coronary artery disease, depression, fibromyalgia/chronic fatigue syndrome, post-traumatic stress disorder (PTSD), anxiety, chronic pain, and hypertension (Rosaura Polak et al., 2015; Thabrew et al., 2018; Fahrenkamp and Benore, 2019; Taghizadeh et al., 2019; Reneau, 2020; da Costa Maynart et al., 2021; Vital et al., 2021).
- Neuromuscular biofeedback: provides a measure of muscle tension with surface electromyography (sEMG) and is used to treat fibromyalgia/chronic fatigue syndrome, repetitive strain injury, tinnitus, arthritis, diabetes, pediatric migraine, anxiety, chronic pain, temporomandibular junction disorders, and adult tension headache (Blume et al., 2012; Šečić et al., 2016; Baumueller et al., 2017; Lazaridou et al., 2023).
- Skin conductance biofeedback: evaluates the activity of eccrine sweat glands and is used in the treatment of anxiety, hypertension, and car/seasickness (Elavally et al., 2020).
- Peripheral skin temperature biofeedback: measures the temperature of the fingers and/or toes and is used to treat repetitive strain injury, arthritis, diabetes, migraines, anxiety, chronic pain, hypertension, and Raynaud’s disease (Fiero et al., 2003; Karavidas et al., 2006; Fritsche et al., 2013).
- Biofeedback of the Central Nervous System (or neurofeedback) which will be described in the next paragraph.

Biofeedback can include implicit or explicit information (Kuikkaniemi et al., 2010; Nacke et al., 2011). In the explicit mode, the feedback (visual, auditory or in some cases tactile) is provided directly to the subject so that he himself acts on the biosignal to be regulated; feedback then acts as a direct correlate of this biosignal. Instead, in the implicit mode, the feedback is not explicitly presented to the subject, but generates changes in one or more details of the experimental condition. Therefore, the subject is not directly aware of his biosignal, but obtains implicit access to a correlate of this biosignal as the latter changes the behavior of the system that the subject is observing (e.g., a video game whose content is changes based on the subject’s heart rate). Since the studies considered in this review used heart rate variability (HRV) and peripheral skin temperature biofeedback, we will briefly describe only these two modalities.

### 2.1 Peripheral skin temperature

Peripheral skin temperature biofeedback measures changes in skin temperature, particularly in the extremities, such as the hands and feet. This form of biofeedback is based on the principle that changes in skin temperature are linked to changes in the autonomic nervous system (ANS), which controls many of the body’s involuntary processes. The peripheral temperature depends on the diameter of the arterioles, the small blood vessels that transport blood to the periphery of the body and whose walls contain smooth muscle tissue innervated by the nerves of the sympathetic nervous system. When the sympathetic system is activated (e.g., due to a stress response), a release of norepinephrine occurs, which stimulates the alpha-adrenergic receptors of the smooth muscle tissue of the blood vessels with consequent narrowing of the latter. When blood vessels narrow, blood flow decreases and, consequently, so does skin temperature. Since there is no parasympathetic innervation (e.g., a relaxation response) of the blood vessels (i.e., the parasympathetic nervous system cannot act directly on the blood vessels to cause vasodilation), in order to allow their dilation and, therefore, increase in peripheral temperature, it is necessary to decrease the activity of the sympathetic nervous system, consequently decreasing the amount of norepinephrine that binds to alpha-adrenergic receptors. The subject must then learn to “turn off” the sympathetic response in order to increase the temperature of the fingers (Freedman et al., 1983; Peper et al., 2009). During skin temperature biofeedback, sensors are placed on the fingers or toes to measure skin temperature. A computer program then provides visual, auditory or sometimes tactile feedback on temperature changes, with the aim of teaching the subject to increase or decrease the skin temperature at will, voluntarily fibromyalgia.

### 2.2 Heart rate variability

Heart rate variability (HRV) refers to changes in the time interval between two consecutive heartbeats (Shaffer and Ginsberg, 2017). HRV reflects the balance of the ANS (Shaffer et al., 2014) and corresponds to the rhythmic accelerations and decelerations of the heartbeat. The heart rate accelerates (i.e., increases) when R-R intervals (i.e., the intervals between beats) shorten and decelerates (i.e., decreases) when R-R intervals lengthen (Berntson et al., 1997). These accelerations and decelerations are also referred to as heartbeat oscillations. The amplitude and complexity of these oscillations are an indicator of the body’s ability to self-regulate and, according to Lehrer (2007). HRV constitutes a necessary component of the negative feedback mechanism that regulates heart rate and blood pressure. When blood pressure increases, baroreceptors (receptors specialized in sensing pressure within blood vessels) detect this increase and send a signal for the heart rate to slow down, leading to a decrease in blood pressure. However, when blood pressure decreases, the baroreceptors send a signal to increase the heart rate and therefore raise blood pressure. Both heart rate and blood pressure fluctuate continuously to maintain homeostasis, the physiological balance of the body. The baroreceptor reflex therefore constitutes a fundamental component of HRV. The second component is represented by respiratory sinus arrhythmia. HRV oscillations reflect the interaction between the sympathetic and parasympathetic branches of the ANS (Acharya et al., 2006). The sympathetic branch increases heart rate, while the parasympathetic branch decreases it. This phenomenon constitutes respiratory sinus arrhythmia, which refers to the fluctuation in heart rate that accompanies breathing: the heart rate increases with inhalation (which activates the sympathetic portion of the ANS) and decreases with exhalation (which activates the parasympathetic portion of the ANS). HRV biofeedback works in a similar way to skin temperature biofeedback but, unlike the latter, provides real-time feedback (visual, auditory, or tactile) of the subject’s heart rate variability which, in this way, can learn to achieve control of specific cardiorespiratory processes.

### 2.3 Electromyography

Surface electromyography (sEMG) consists of measuring the electrical signal produced by muscles during contraction. The source of the electrical signal is the muscle action potential (MAP). MAPs are produced by motor units, i.e., the “building blocks” of skeletal muscle fibers. Each motor unit consists of a motor neuron in the spinal cord, the muscle fibers it innervates, and an axon that transmits electrical signals from the neuron to the muscle fibers. Once the neuron sends the electrical signal to the axon, it releases the neurotransmitter acetylcholine, which stimulates the muscle to contract. This communication process between the spinal cord and the muscle is achieved through the MAP, i.e., a change in the electrical charge of the muscle cell membrane from a resting value of approximately −70 mV to a peak of approximately +30 mV, the potential d‘action. The amplitude of the signal that the biofeedback equipment collects from the muscles depends on the number of action potentials generated, the firing rate of each active neuron and the amount of fatty tissue between the electrode and the muscle. The more adipose tissue between the muscle and the electrode, the smaller the recorded signal will be. Since the amplitude of the recorded sEMG signal depends on the number of active neurons, the innervation rate (i.e., the number of muscle fibers controlled by a single motor neuron) plays an important role in determining how large the amplitude of the sEMG signal will be. Motor units responsible for large, powerful movements innervate a greater number of muscle fibers, while those responsible for fine movements innervate fewer muscle fibers. The greater the number of muscle fibers that the neuron controls, the greater the innervation rate, the more powerful the movement produced by that muscle and the greater the amplitude of the sEMG signal. As regard the placement of the electrodes during sEMG biofeedback, once the muscles being trained have been identified, it is necessary to decide whether to use a narrow or wide electrode placement. sEMG records electrical activity from muscles between two active electrodes and, as highlighted by Sherman (2003), the electrical signal that triggers muscle contraction in one area also influences electrical activity in adjacent areas. Therefore, the electrical activity generated by muscles underlying and adjacent to the target muscle could also be recorded. Therefore, the wider the positioning of the active electrodes, the greater the number of muscles whose signal will be acquired and the greater the amplitude of the sEMG signal recorded and shown. Narrow placement (approximately 2 cm or less between active electrodes) is appropriate when you want to work with a specific muscle, minimizing cross talk between muscles, i.e., signals from adjacent or underlying muscles. A wider positioning, however, is appropriate for the relaxation of large groups of muscles, as you want to obtain as much information as possible regarding the muscular activity in the area of interest. Finally, in relation to the steps to follow for sEMG biofeedback training, after having identified the target muscle or muscles, we move on to positioning the electrodes based on the type of training you want to carry out. For example, if you want to isolate the activity of a target muscle, you place one set of electrodes on the muscle of interest and the other set on the muscle that co-contracts unnecessarily together with the muscle of interest. At this point the subject is asked to increase the tension in the muscle of interest and to observe what happens to the muscle that co-contracts. The purpose of this training is to teach the subject to activate the target muscle and to reduce the activation of the co-contracting muscle, through the use of real-time auditory or visual feedback (for example, the sEMG signal shown on the monitor screen computer) through which the subject, over the course of several sessions, learns to control his own muscle activation.

## 3 Neurofeedback

Neurofeedback (NF) is a form of biofeedback based on characteristics of brain activity. This is a technique that has been extensively studied over the last few decades and whose foundations have been laid since the 1930s. In fact, in the middle of these years, some groups of researchers trained subjects to block alpha waves through a classical conditioning mechanism (Durup and Fessard, 1935; Loomis et al., 1936). Subsequently, in a 1941 study, Jasper and Shagass (1941) demonstrated, for the first time, that the alpha rhythm could be suppressed voluntarily, thus bringing to light the concept of “voluntary control” of electroencephalographic activity. For the use of operant conditioning to modulate EEG activity (i.e., using the information derived from the EEG as a reward to be provided to the subject in real time) we had to wait until the 1960s (Arns, 2010; Sterman et al., 2010; Kamiya, 2011). Since that time this technique has been increasingly used for the treatment of various neuropsychiatric conditions (ADHD, epilepsy, stroke, developmental disabilities, migraines, insomnia, etc.), definitively establishing the birth of what we know today as Neurofeedback. In 2011, Hammond describes seven types of neurofeedback, which can be used to treat different conditions:

1. The frequency/power NF (or EEG-NF): modifies the amplitude or speed of specific frequency bands within specific brain regions. For this purpose, electrodes placed on the surface of the subject’s scalp are typically used and it is a technique classically used in the treatment of ADHD, learning disorders, anxiety, insomnia and other pathological and non-pathological conditions (for example, in improving sports performance; Salimnejad et al., 2019; Gong et al., 2021).
2. The Slow Cortical Potential NF (SCP-NF): modifies the direction (positive or negative) of slow cortical potentials and is used to treat ADHD, migraines, and epilepsy (Christiansen et al., 2014).
3. The Low-Energy Neurofeedback System (LENS): uses a very weak electromagnetic signal to modify the subject’s brain waves while the latter is in a condition of immobility and with his eyes closed (Zandi-Mehran et al., 2014). This technique is considered a passive form of NF and has been used to treat conditions as diverse as head trauma, ADHD, insomnia, fibromyalgia, restless legs syndrome, anxiety, depression, and anger.
4. NF with Hemoencephalography (HEG): provides feedback regarding cerebral blood flow and is used to treat migraine (Dias et al., 2012).
5. The Live Z-Score NF: involves the continuous comparison of several variables related to brain electrical activity (e.g., power, coherence, and asymmetry) with a normative database to provide moment-to-moment feedback and is used to treat insomnia (Collura et al., 2010).
6. Low Resolution Electromagnetic Tomography (LORETA): uses 19 electrodes to monitor phase, power and coherence (Pascual-Marqui et al., 1994) and is used for addictions, depression and obsessive-compulsive disorder.
7. Functional Magnetic Resonance Imaging NF (fMRI-NF): uses the activity of the subcortical areas of the brain as feedback (Lévesque et al., 2006; Hurt et al., 2014).

To understand the functioning of the NF, reference must be made to learning processes, which occur thanks to a brain capacity known as neuronal plasticity (Kolb, 1995; Maren and Baudry, 1995; Rosenzweig and Bennett, 1996), based on neuromodulation and on long-term potentiation (LTP; Abarbanel, 1995). It has been proposed that during NF training, the relevant neural networks are modified through the process of neuromodulation (Evans and Abarbanel, 1999) and that these changes are consolidated through LTP triggered by continuous feedback activity during this training (Evans and Abarbanel, 1999; Sterman and Egner, 2006).

Recent discussions іn the field оf neurofeedback have often centered оn the role оf long-term potentiation (LTP) as a foundational mechanism underlying the efficacy оf NF training protocols (Gabrielsen et al., 2022; Spreyermann, 2022). LTP, characterized by the long-lasting strengthening оf synapses based оn recent patterns оf activity, has been widely recognized for its role іn learning and memory within the neuroscience community. However, the complexity оf neural responses elicited by NF protocols such as Alpha/Theta (A/T) and Infra-Low Frequency (ILF) NF suggests that a broader spectrum оf neuroplastic mechanisms may be at play. Unlike traditional models that primarily focus оn LTP’s role іn synaptic efficacy, A/T and ILF NF protocols exemplify how NF can modulate broader neural networks and states. This modulation does not solely rely оn the strengthening оf synaptic connections but also involves the induction оf desirable brain states and the balance оf neural oscillations across various frequency bands (Muñoz-Moldes and Cleeremans, 2020).

In this review, we will only consider EEG-NFB, as the included articles exclusively used this technique. During NF training, electrophysiological processes provide the basis for information transmission, whereby brain oscillations present in specific cortical areas reflect cognitive processes. These brain oscillations are divided into different frequency bands: delta (0.5–4 Hz), theta (4–8 Hz), alpha (8–13 Hz), beta (15–30 Hz), and gamma (> 30 Hz). In a classic NF set-up, EEG activity is recorded using a referential (or monopolar) montage. During an NF session, a real-time analysis of EEG frequencies is shown to provide information regarding the amplitude of activity within each of the different frequency bands. Once the desired changes in the electrophysiological patterns of interest have been produced, the subject will receive a reward in the form of an auditory, visual or even tactile signal. During numerous training sessions, the participant will learn to develop strategies to consciously and voluntarily modify and self-regulate their brain electrical activity. Neurofeedback therefore constitutes neurocognitive training based on the principles of operant conditioning and over the years has found application in the treatment of various psychopathological conditions and in the improvement of cognitive and motor performance.

There are several protocols that can be used for NF training:

- The alpha protocol consists of the enhancement or inhibition of this frequency band and is used in the treatment of pain and brain trauma, to reduce anxiety and stress and to improve cognitive performance (e.g., memory and functions executive).
- The beta protocol is based on the enhancement of this frequency band, usually in association with the simultaneous inhibition of the theta frequency band, and is used in the treatment of ADHD, epilepsy and autism spectrum disorders and in the improvement of attention and concentration.
- The alpha/theta protocol has the function of enhancing the theta frequency band compared to the alpha band and is used to reduce stress and for the treatment of anxiety and addictions.
- The delta protocol consists of the enhancement or inhibition of this frequency band and is used in the treatment of ADHD and learning disabilities and to reduce depressive and anxious states and pain (e.g., headaches and migraines) and improve the quality of sleep.
- The theta protocol focuses on the enhancement of this frequency band, in particular in the frontal-medial region (frontal-midline theta, FMT) and is used to improve motor performance (e.g., the flow experience) and cognitive performance (e.g., e.g., executive functions).
- The Infra-Low Frequency (ILF) protocol targets the very low-frequency brain waves, often below 0.1 Hz, engaging with the foundational aspects оf brain function. ILF Neurofeedback іs particularly noted for its potential іn stabilizing the nervous system, thereby contributing tо improvements іn emotional regulation, stress reduction, and overall cognitive function. Its application spans a wide range оf conditions, leveraging the deep and often subconscious levels оf neural activity tо facilitate therapeutic change. This protocol exemplifies the advanced frontier оf neurofeedback research, exploring how subtle modulations іn brain activity can yield significant health benefits (Doren et al., 2018).

After discussing the various NF training protocols, such as alpha, beta, delta, and theta protocols, it is pertinent tо delve deeper into the nuanced mechanisms оf action behind these methods. Specifically, Alpha/Theta Neurofeedback and Infra-Low Frequency (ILF) Neurofeedback diverge from these protocols by focusing оn state feedback rather than learning reinforcement. Alpha/Theta Neurofeedback іs designed tо induce a relaxed оr meditative state, enhancing therapeutic outcomes through the modulation оf brain states. Similarly, ILF Neurofeedback targets the autonomic nervous system, aiming tо achieve a balanced physiological state. This delineation underscores the unique therapeutic potential оf these modalities beyond the classical operant conditioning frameworks, highlighting their distinct approach tо facilitating therapeutic change. Alpha/Theta Neurofeedback aims tо induce a relaxed оr meditative state, enhancing therapeutic outcomes through state feedback rather than learning reinforcement traditionally associated with neurofeedback. Similarly, ILF Neurofeedback focuses оn modulating the autonomic nervous system tо promote a balanced physiological state. This distinction highlights the unique mechanisms оf action for these neurofeedback modalities, underscoring their therapeutic potential beyond classical operant conditioning frameworks.

Building оn the discussion оf various neurofeedback (NF) training protocols, іt іs crucial tо address the potential for personalization within these therapeutic modalities. The concept оf tailoring NF procedures tо individual characteristics, as exemplified by M. Arns with Q-based NF and the symptom and state change-based individualization pioneered by the Othmer group, represents a significant advancement іn the field. This approach underscores the importance оf adapting NF protocols tо meet the unique neurophysiological and psychological profiles оf each individual, potentially transforming non-responders into responders.

The variability іn response tо standard NF protocols highlights the necessity for a more nuanced approach. Personalization can involve adjusting the training parameters based оn quantitative EEG (qEEG) analyses, as done іn Q-based NF, оr modifying protocols based оn symptomatic and state changes observed during the training process. Such individualized strategies not only cater tо the specific needs оf the participants but also optimize the chances оf therapeutic success.

The results of NF training, however, are not always consistent. There is, in fact, a significant part of the population subjected to this treatment who does not seem to benefit from its effects and who does not achieve the required voluntary control of the frequency bands covered by the training, the so-called non-responders (or non-performers or non -regulators; Weber et al., 2010; Alkoby et al., 2018). In this regard, two categories of predictive factors of the ineffectiveness of NF training have been identified: psychological predictors and neurophysiological predictors. Regarding the former, Witte et al. (2013) showed that subjects’ beliefs regarding their ability to control technological devices can predict performance in NF training, while Kober et al. (2013) found an influence of the mental strategies used by subjects to control the bars that reflect their brain activity in real time. The authors found that the mental strategy used in NF sessions depended on the brain parameter chosen for training. For example, for the SMR protocol the participants who had not used mental strategies showed a better performance, while for the gamma protocol the mental strategy adopted showed no influence on performance. As regard neurophysiological predictors, Wan et al. (2014) showed that alpha wave amplitude in resting condition before NF training significantly correlated with success in EEG learning. Also confirmed by other studies (e.g., Kotchoubey et al., 1999; Neumann and Birbaumer, 2003; Weber et al., 2010), this observation has led to considering individual differences in the amplitude of the different frequency bands as a factor not to be underestimated within NF training. What we therefore asked ourselves is whether a personalization of NF procedures, in which the characteristics of the training are adapted to the individual, can transform non-responders into responders. For example, two different studies (Bauer, 1976; Nan et al., 2012), both aimed at improving performance in the digit span task following NF training aimed at the alpha frequency band, obtained opposite results: Bauer (1976), who used a fixed alpha band width, did not obtain improvements in subjects’ performance; while Nan et al. (2012), who used the individual alpha frequency (IAF; that is, they calculated the alpha band for each participant), observed improvements in the subjects in the same test used by Bauer. This demonstrated the need to adapt NF training to the specific characteristics of each subject’s brain electrical activity. A second property to check is the type of feedback to use (visual, auditory, or tactile). For example, Mathiak et al. (2015) studied the effect of using “social” feedback (a smiling avatar) on the ability to control an EEG frequency during NF training. The authors showed that social feedback produced greater activation in the anterior cingulate cortex and reward-related circuits compared to standard feedback (a moving bar). Furthermore, the use of this type of feedback improved the subjects’ ability to control the frequency bands trained during the NF procedure. Therefore, using a type of personalized feedback, based on the subject’s characteristics, could lead to a better performance in the training. Finally, the last characteristic that must be taken into consideration is the type of mental strategy to employ. For example, Nan et al. (2012) showed that thinking about pleasant situations or people (e.g., thinking about family and friends) represents an efficient mental strategy to enhance the alpha frequency band, while Hardman et al. (1997) and Kober et al. (2013) showed that the best way to make NF training effective, respectively, at slow cortical potential (SCP) and sensorimotor rhythm (SMR), is to not use any mental strategy. Therefore, customizing the type of mental strategy based on both the type of NF protocol and the individual’s characteristics could lead to better performance in NF training.

## 4 Clarification оn the relationship and integration оf BF and NF techniques

In the context оf our review, іt іs essential tо delineate the interrelation between BF and NF. While both modalities leverage the principle оf feedback for self-regulation, their application domains and mechanisms оf action differ. BF techniques facilitate control over various physiological processes, potentially including but not limited tо cardiovascular, respiratory, and musculoskeletal systems. In contrast, NF exclusively focuses оn modulating brain functions, offering a targeted approach tо influence cognitive and psychological states.

It іs crucial tо note that the integration оf these techniques can be implemented either іn parallel оr sequentially. In a parallel approach, BF and NF are applied simultaneously during the same treatment session, allowing the patient tо receive feedback оn both peripheral physiological parameters (such as heart rate variability оr muscle tension іn the case оf BF) and brain activity (such as brain waves іn the case оf NF). This approach aims tо maximize the treatment’s effectiveness by synergizing the effects оf both techniques. Alternatively, a sequential approach involves applying NF and BF іn separate sessions, with the order оf administration varying (first NF then BF, оr vice versa). This method can be particularly useful for addressing specific patient needs, allowing for a greater focus оn areas оf intervention identified at different stages оf the treatment. Both approaches have the potential tо offer unique benefits, and the choice between parallel оr sequential application should be guided by the specific needs оf the patient, the conditions being treated, and the therapeutic objectives defined by the practitioner. Future research should further investigate the conditions under which each approach may be more advantageous, thus contributing tо greater customization and effectiveness оf treatments integrating BF and NF.

Our exploration into the combined use оf BF and NF іs predicated оn the hypothesis that integrating these techniques can harness their respective strengths, potentially amplifying therapeutic and performance outcomes. By concurrently addressing the CNS and PNS, the integrated approach aims tо promote a more comprehensive state оf self-regulation, enhancing both mental and physical well-being. Our review seeks tо contribute new insights into how this synergistic application оf BF and NF might differ іn effectiveness from using multiple BF techniques іn isolation. By examining studies that implement this integrated methodology, we aspire tо uncover distinct benefits that could redefine best practices іn biofeedback applications for therapeutic and performance enhancement purposes.

## 5 The integrated BF + NF approach

In the following paragraphs, we will examine the effectiveness of an integrated BF + NF training based on the results present in the literature.

### 5.1 Nicotine addiction

According to a study published in 2021 in The Lancet, there were an estimated 1.4 billion smokers worldwide in 2019 (Reitsma et al., 2021). Smoking impacts all organs of the body and is the cause of numerous premature deaths and diseases (especially oncological and cardiovascular diseases; US Preventive Services Task Force, Owens et al., 2020). Chronic nicotine use has been associated with both functional and structural impairments of the Central Nervous System (Pomerleau, 1992; Musso et al., 2007; Goriounova and Mansvelder, 2012; Fedota and Stein, 2015; Sutherland et al., 2016) and several studies have reported atrophy of white and gray matter especially in the frontal, prefrontal, temporal, fronto-parietal areas and in the cingulate and cerebellar cortices (Brody et al., 2004; Gallinat et al., 2006; Swan and Lessov-Schlaggar, 2007; Kühn et al., 2012). Networks involved in cognition and executive functions, such as the Default Mode Network (DMN), have been shown to be affected by addiction (Hong et al., 2009; Weiland et al., 2015). The prefrontal networks involved in attention show a decrease in activity at least partly related to the duration of nicotine addiction (Musso et al., 2007) and a disorganization of the network topology characterized by a decrease in the efficiency of the DMN has been shown and other brain networks (Lin et al., 2015). These CNS alterations, which also include increased resting connectivity in prefrontal areas involved in reward circuits (Janes et al., 2012), may help explain, at least in part, why interventions aimed at extinguishing reward addiction nicotine continue to be difficult or even ineffective (Ashare et al., 2014). Furthermore, the greatest challenge in nicotine addiction cessation programs consists in the prevention of relapses, which are triggered, among other things, by negative affective states and conditions related to anxiety and stress, smoking being often used as a means to deal with these situations (Pandria et al., 2018). Therefore, ex-smokers need alternative coping strategies (Brandon et al., 1990; Crevenna, 2010; Pandria et al., 2020).

In addition to pharmacological interventions, whose side effects however constitute the main reason for their discontinuation (Williams et al., 2007; Halperin et al., 2009), alternative approaches have also been used to address substance addiction (nicotine included), such as behavioral therapies and biofeedback and neurofeedback programs. BF and NF training have been shown to be effective in the management of anxiety disorders, depression and stress-related conditions (Hammond, 2005; Gruzelier et al., 2014; Tabachnick, 2015) and have been used since the 1970s, in the treatment of addictions (Lamontagne et al., 1977; DeGood and Valle, 1978).

For instance, given the significant challenge posed by alcohol dependence, particularly with relapse rates reaching up tо 80%, the integration оf neurofeedback (NF) and biofeedback (BF) іn treatment protocols offers a promising avenue for intervention. Although some addiction facilities have begun tо implement NF/BF approaches, the current deployment іs not commensurate with the high prevalence and relapse rates associated with alcohol dependence. This disparity underscores a critical need for expanding and intensifying the use оf NF/BF іn addiction treatment programs.

The early work оf Penniston іn the realm оf Alpha/Theta (A/T) neurofeedback for alcohol dependence highlights the potential оf such personalized interventions. Initially utilizing thermal and Skin Conductance Level (SCL) feedback, Penniston’s approach evolved tо incorporate NF techniques, setting a foundational precedent for integrating various biofeedback modalities іn treating addiction (Peniston and Kulkosky, 1989). This multidimensional feedback strategy not only addresses the physiological aspects оf addiction but also engages deeper psychological patterns associated with relapse.

These approaches have been shown to be encouraging in facilitating the self-regulation of relapse-related factors, such as craving and stress (Canterberry et al., 2013; Hanlon et al., 2013; Li et al., 2013; Kim et al., 2015; Hartwell et al., 2016; Bu et al., 2019) and can positively influence the degree of nicotine dependence and the presence of psychiatric symptoms (Pandria et al., 2018).

In a recent 2023 study, Pandria and colleagues used an integrated BF + NF approach to evaluate its effects on extinguishing nicotine addiction. The intervention aimed to combine an increase in vasodilation through a BF training of peripheral skin temperature (Tolin et al., 2020) and a facilitation of a state of deep relaxation through a NF training targeted at the alpha and theta (Gruzelier, 2009, 2014a,b,c). The BF training of skin temperature was chosen as this constitutes a reliable marker of stress which, as previously mentioned, plays a primary role in nicotine addiction (Kassel et al., 2003; Tsourtos et al., 2008) and it is also influenced by nicotine itself through its stimulatory action on the sympathetic system, causing peripheral vasoconstriction (Benowitz and Burbank, 2016). The alpha-theta NF protocol was instead chosen as it has been successfully applied in other forms of addiction (Peniston and Kulkosky, 1989; Masterpasqua and Healey, 2003; Scott et al., 2005; Sokhadze et al., 2008), in PTSD (Peniston and Kulkosky, 1991) and in improving mood (Raymond et al., 2005).

The study participants (17 smokers) underwent a multi-phase intervention. The baseline evaluation (baseline, T0) consisted of a first clinical and behavioral examination and a second electrophysiological and neuropsychological investigation. Subsequently, five BF training sessions were administered and, immediately afterwards, the participants underwent an intermediate evaluation (T1), at the end of which the alpha-theta NF protocol was administered, divided into 20 sessions. Finally, participants underwent the last survey (T2). The results of this integrated study showed a reduction in participants’ exhaled carbon monoxide (CO) levels and a decrease in total oxidative stress (TOS) levels. Smoking in fact leads to an increase in oxidative damage (Kamceva et al., 2016; Karademirci et al., 2018) which leads to the generation of free radicals and inflammatory responses (Csordas and Bernhard, 2013; Messner and Bernhard, 2014) and has been shown that there is a correlation between oxidative stress and the number of cigarettes smoked (Kamceva et al., 2016). From these results we could suggest a protective role of BF + NF training against the oxidative load induced by smoking, as the participants in the study by Pandria et al. (2023) who were highly dependent on nicotine still managed to achieve a significant reduction in oxidative stress levels. Smoking also causes dysregulation of hormonal responses to stress (Childs and de Wit, 2009), leading to the establishment of abnormal anxiety thresholds. As argued by Kalantzi-Azizi and Degleris (1992) and Parrott (1998), the variation in mood during both long and short periods of abstinence leads to conditioned learning, whereby abstinence and other negative affective states can be interpreted as stressors that lead to the act of smoking which, in turn, alleviates withdrawal symptoms and negative affective states, creating a vicious circle. Therefore, as argued by Pandria et al. (2018), stress constitutes a central element in nicotine addiction. In the study examined for this narrative review, positive changes were observed in relation to stress, as a reduction in the scores in the State–Trait Anxiety Inventory was noted, in particular in the items relating to trait anxiety, confirming what Taylor et al. (2014) claimed namely that quitting smoking reduces anxiety, stress and depressive symptoms, improving mood. BF + NF training has also proven effective in improving self-esteem, particularly in women and in participants with a strong nicotine addiction, subjects who, according to some studies, are characterized by a low level of self-esteem (Croghan et al., 2006; Guillon et al., 2007). Although a negative correlation between self-esteem and smoking has not yet been demonstrated (Szinay et al., 2019), according to Freijy and Kothe (2013) high levels of self-esteem lead to a greater likelihood of quitting smoking.

In addition, BF + NF training produced positive results in improving inhibitory control, visual attention, task switching, and working memory, assessed through the Stroop test, Trail A test, and Digit Span, respectively. Although some studies believe that nicotine promotes cognitive improvement through stimulation of brain areas related to cognition (Couey et al., 2007; Kenney and Gould, 2008; Wallace and Bertrand, 2013; Kutlu and Gould, 2015), there are no there is still clear evidence to support this thesis, as the cognitive improvement could simply be given by the relief from withdrawal symptoms (Heishma et al., 1994; Heishman, 1998). Finally, this study showed neuroplasticity capacity in the group of smokers subjected to integrated training, which showed increased synchronization especially between the networks involved in cognitive control (frontoparietal network, FPN), in goal-directed behavior (DMN) and in visual processing (visual network, VIS). The increased synchronization between the visual network and other resting-state networks (RSNs) is considered an indicator of better integration of sensory information (Dobrushina et al., 2020). Finally, Modi et al. (2015) found that trait anxiety is associated with reduced connectivity between the DMN and the VIS, thus the reduction in STAI trait anxiety scores observed in the study by Pandria et al. (2023) it may reflect the increase in connectivity observed in these two networks.

The effectiveness of EEG-NF training in the treatment of nicotine addiction has also been documented by recent reviews (Pandria et al., 2020; Keilani et al., 2022). Griffith and Crossman's (1983) study used an NF intervention targeting the alpha frequency band and showed that, while smoking a cigarette, all smokers had decreased occipital alpha activity, and immediately after smoking, five smokers of six showed a continuous increase in heart rate and four a decrease in skin temperature. Furthermore, during the NF training, four out of six smokers managed to increase their alpha activity, two participants stopped smoking at the end of the 6-month follow-up period and the other four reduced their daily number of cigarettes. The study by Bu et al. (2019) also showed a notable decrease in the number of cigarettes smoked per day, as well as a significant improvement in craving scores and a significant reduction in the amplitude of the P300 event-related potential (ERP), related to craving and smoking (Littel and Franken, 2011; Evans et al., 2013). The review by Pandria et al. (2018) also took into consideration the study by Szalai et al. (1986), which used an alpha frequency band modulation protocol and divided the participants into 10 deprived smokers (DS) and in 10 non-deprived smokers (NDS) including, as active controls, ex-smokers (EF), and non-smokers (NS). This study showed that deprived smokers and non-smokers were able to reduce the amplitude of the alpha frequency band during a backward memory task, while deprived smokers were able to reduce the alpha frequency band even during the alpha suppression condition “alpha rhythm.” Furthermore, only non-smokers were able to increase alpha amplitude during the alpha rhythm-enhanced condition, while non-deprived smokers showed a significant reduction during this task.

However, regarding the effectiveness of peripheral skin temperature BF in the treatment of nicotine addiction, the study by Pandria et al. (2018), reviewed by Pandria et al. (2020) and by Keilani et al. (2022), used finger temperature as BF training with the aim of having participants gain control over ANS functions. The results showed positive effects of BF on the ability to control skin temperature and a significant improvement (in males, but not in females) in the degree of nicotine dependence, measured with the Fagerström test, and in the score of the General Health Questionnaire. Furthermore, a reduction in the presence of psychiatric symptoms, anxiety, depression, and withdrawal symptoms was observed, while an increase in self-esteem was observed. Finally, a reduction in the number of participants with moderate and severe addiction was noted. The second study taken into consideration in the review by Pandria et al. (2020) is that of Grimsley (1990), which aimed to compare the ability to increase the skin temperature of the hand between smokers and non-smokers and used three groups of subjects: smokers who smoked before the BF session (SS), smokers who did not smoke before the session (SNS), and non-smokers (NS). This study demonstrated how smoking influences the ability to control skin temperature, as the NS and SNS groups were able to increase their skin temperature, while the SS group not only did not have this ability, but also showed a slight decrease of skin temperature after the BF session. Grimsley therefore argues that smoking causes lower hand skin temperature due to vasoconstriction and hinders the ability to modulate it, influencing the results of BF sessions and other psychological treatments involving relaxation.

Although the reviews taken into consideration present limitations, both at the level of the included studies (for example: methodological limitations such as the lack of a control group, a sample consisting of few subjects or non-standardized clinical outcomes) and at the level of the reviews themselves (for example: a small number of studies included and a poor quality of the same), the results show that these alternative approaches constitute promising measures to help smokers to quit smoking, facilitating the modulation of the activity of the Central and Autonomic Nervous System. Similarly, the study by Pandria et al. (2023) confirms what emerged from the reviews examined, demonstrating the effectiveness of an integrated BF + NF approach on the clinical, behavioral, and cognitive aspects of smokers. At present, it is not possible to determine whether this integrated approach produces better results than the two approaches applied individually, as there are no effect size measures either in the reviews considered or in the study by Pandria and collaborators. Furthermore, more studies, greater methodological rigor and follow-ups at multiple time intervals are needed to be able to carry out a precise analysis of the effectiveness of these techniques.

Recent studies have emphasized the importance оf stress modulation techniques as crucial components іn behavioral therapies, highlighting the potential оf suicidal thoughts tо temporarily reduce stress through the decrease оf heart rate variability (HRV) and skin conductance response (SCR), as demonstrated by Linehan et al. (2015). This suggests a calming effect оf dysfunctional covert actions (suicidal thoughts), raising significant questions about the efficacy оf targeted therapeutic strategies, such as paced breathing taught іn dialectical behavior therapy. However, іt іs critical tо note that such techniques have traditionally been taught without the integration оf BF/NF techniques, specifically mentioning the absence оf a combined NF + BF approach. This observation opens new avenues for future research, suggesting that the integration оf NF and BF could offer an innovative intervention strategy tо enhance stress management and reduce the incidence оf suicidal thoughts. Our current study identifies and highlights the need tо further explore this hypothesis through methodologically rigorous studies, іn order tо assess the efficacy оf an integrated therapeutic approach combining BF and NF tо effectively address stress-related dysfunctions.

### 5.2 Sports performance

In the sporting world, psychophysiology provides valid support for improving the performance aspects of an athlete, who often finds himself facing races and competitions. The evaluation of performance aspects is limited if these are considered exclusively or attesting to physiological and mental parameters. Mind and body are one, they appear as two sides of the same coin that interact in the various dynamic processes studied and explored in depth in the various interdisciplinary fields that have long sought to provide answers on the body–mind combination (Cacioppo et al., 2007). In recent years, the psychophysiological monitoring of this combination has grown exponentially, thanks to the growing awareness that athletes and coaches are continuously searching for alternative and innovative methodologies for enhancing physical and mental abilities that can be positively correlated with sports performance (Diotaiuti et al., 2021a,b, 2023; Siekańska et al., 2021; Hsieh et al., 2023). Athletes, having to manage the times and methods of execution of the technical/athletic gesture, look for programs and technologies that accurately and thoroughly improve their performance (Lange-Smith et al., 2023). An example is given by sports such as archery or shooting where the management of arousal turns out to be a fundamental aspect for dealing with physiological and behavioral conditioning coming from internal and external responses which influence performance (Marzbani et al., 2016; Mikicin et al., 2018; Diotaiuti et al., 2021c).

The evolution of research has led to the identification of a way to monitor and intervene on these aspects: biofeedback and neurofeedback training. In the sports field, the first studies were conducted using biofeedback and date back to 1991, when Petruzzello and colleagues were the first to investigate the use of this technique in this area. At the beginning, the studies did not produce significant effects due to various problems, attributed by the researchers to exploratory interventions and the lack of preliminary knowledge of the basic levels of EEG, HR and respiration (Petruzzello et al., 1991). Over the years, the exploratory and interventional phenomenon of BF has extended to new protocols, highlighting its real effectiveness in physiological processes such as heart rate variability and muscle tension. Training aimed at these aspects can increase the effects of personal awareness to reduce and control states of stress (De Witte et al., 2019). A classic example is deep breathing used to lower the heart rate, inducing a state of relaxation during high-pressure moments in a competition (Russo et al., 2017; Lalanza et al., 2023). Over time, in addition to the BF technique, the application of NF has also begun to take hold in the field of sports performance, through which the athlete learns to modulate his own brain activity by trying to achieve the desired states associated with a greater control of attention during performance.

The research conducted using the NF has mainly focused on the study of athletes belonging to sports disciplines that require attention and concentration skills. In this regard, some authors have been able to verify the effectiveness of NF training on the brain activity of some golfers. Athletes were trained to self-regulate their brain functions, particularly theta waves of the medial frontal region associated with cognitive and attentional control (Chen et al., 2022; Corrado et al., 2024). Recently, Rydzik et al. (2023) highlighted that the effect of sports training is positively associated with the NF method which, if used by athletes during training, can lead to the control and improvement of psychophysiological aspects. However, the author himself does not hide the limitations of the research by inviting the production of further research aimed at verifying the effectiveness of other protocols that would lead to better results in sporting contexts and with individual athletes.

Despite the considerable presence of NF studies, there are many difficulties faced by researchers in verifying the effectiveness and reliability of the various types of training proposed in sport (Vernon, 2005).

In a recent review on the topic, Gong et al. (2021) examined the experiences and points of view of practitioners carrying out NF training in sport. The authors have brought to light many controversies related to the technical aspects and application areas of the various NF protocols adopted. There are many studies conducted with BF and NF training, but little evidence has been shown on the effectiveness of combined scientific protocols. If integrated, neurofeedback and biofeedback can provide a valid contribution to the athlete’s performance (Shaw et al., 2012). By monitoring brain activity and physiological responses, athletes can learn to regulate their emotions, manage fatigue, and achieve an optimal state of performance (Dupee et al., 2016). Other evidence has emerged in the rehabilitation field: NF and BF techniques can in fact also be used in rehabilitation from injuries. In this case, neurofeedback can help athletes retrain and recover brain functions affected by concussions or other traumatic brain injuries (Conder and Conder, 2014). Furthermore, it has been seen how biofeedback can help monitor and manage physiological responses during rehabilitation exercises (Giggins et al., 2013). This confirms the hypothesis according to which the combination of neurofeedback and biofeedback protocols can provide a valid contribution to the improvement of mental and physical states, even if they are carried out separately (Sime, 2003). This approach is very close to the theory of Neural Synergy Systems, which explains brain functionality as a complex system, with multiple components and networks that work together to produce coherent and integrated behaviors. This theory supports the idea that the brain operates through the coordination and cooperation of different neural systems rather than individual isolated parts. In their studies conducted with clinical cases, Thompson et al. (2010) confirmed the need to proceed with an integrated biofeedback and neurofeedback approach. Both draw from Porges’ Polyvagal Theory, which provides theoretical aspects to understand the role that some nervous pathways have in relation to the physiological and psychological responses connected to each other. It has been shown that BF can train individuals to vary heart rate, which is an indicator of vagal tone and overall autonomic flexibility (Jimenez Morgan and Molina Mora, 2017; Mosley and Laborde, 2022). Similarly, NF can lead to an improvement in vagal tone, providing individuals with strategies to self-regulate their brain waves and promoting emotional and social well-being (Villamil et al., 2019). In our comprehensive review оf biofeedback and neurofeedback applications, іt іs crucial tо highlight the groundbreaking contributions оf Michael and Lynda Thompson іn the field. Thompson’s research, which elucidates the correlation between the sensorimotor rhythm (SMR) envelope (and thus power) and the outbreath during heart rate variability (HRV) biofeedback, marks a significant advancement іn our understanding оf the physiological underpinnings оf biofeedback interventions (Thompson and Thompson, 2009). This correlation underscores the intricate relationship between neurological processes and respiratory patterns, offering novel insights into the design and optimization оf biofeedback protocols for various therapeutic outcomes. The gap іn the literature regarding studies that combine end-tidal carbon dioxide (etCO2) measurements with neurofeedback (NF) іs noteworthy and points tо an exciting frontier for research, exploring the intersections оf respiratory, cardiovascular, and neurological biofeedback tо deepen our understanding оf physiological coherence and its implications for health and disease. Thompsons’ extensive work, incorporating electromyography (EMG) biofeedback as a consistent component оf their interventions, has contributed tо a more holistic approach tо biofeedback therapy. Their integrative methodology, emphasizing the simultaneous application оf neurofeedback and EMG biofeedback, has been instrumental іn addressing complex clinical presentations (Thompson and Thompson, 2023).

Given the connection and the aspects that the two interventions have in common, it would be reasonable to think that the combined use of the two protocols could produce better effects than their separate application. Most studies conducted in sports have used the two interventions separately (Rijken et al., 2016).

In 2021, Shokri and collaborators compared the integrated BF + NF approach with BF alone on a sample of 45 novice basketball players. The authors randomized the sample into three intervention, two experimental, and one control groups. In the first experimental group the sample performed only 24 biofeedback sessions, while the second experimental group received a combination of neurofeedback and biofeedback. Athletes were monitored in four basic technical skills: lay-ups, chest passes, dribbling, and free throws. Participants in the combined intervention group showed significant improvement in all four core skills, while the biofeedback-only group showed improvements only in layup and passing performance. In each of the four skills measured, attention, reaction time, and vigilance play a decisive role so that, compared to the group that received only the BF, the level of performance was better in the group that received the intervention integrated, confirming its effectiveness. This improvement can be attributed to the changes produced in the brain by neurofeedback, which can lead to a concrete increase in attention and a reduction in reaction times (Shokri and Nosratabadi, 2021).

The results of these two recent studies are clear and evident and show the effectiveness of the double intervention. Future research in sports should focus on protocols that include integrated intervention training. If in the past studies on sport have focused on a separate use of BF and NF, today the integrated approach can provide more possibilities for intervention, integrating multiple aspects to be strengthened. By providing both psychological and physiological training, concrete and effective results can be achieved.

### 5.3 Attention deficit hyperactivity disorder

In recent decades, advances in neuroscience have led to a greater understanding of the functioning of the brain and its dysfunctions associated with disorders such as ADHD (Leffa et al., 2022). Attention Deficit Hyperactivity Disorder (ADHD) is characterized by a pattern of inattention and/or hyperactivity-impulsivity that interferes with functioning or development. It is considered one of the most common psychiatric disorders in children and adolescents (Fawns, 2021) and leads to problems in school, impaired social skills and poorer adaptive functioning in major life activities (Gevensleben et al., 2009). ADHD causes cognitive deficits especially in reference to executive functions, which allow the production of behaviors aimed at achieving a specific goal (Martínez et al., 2016) and is also characterized by excessive sensitivity to reinforcements, which corresponds to a difficulty in waiting for gratification. One of the executive functions most affected in ADHD is inhibitory control, that is, the inability to inhibit irrelevant responses (Barkley, 2022). People with ADHD have slower response times during cognitive tasks (Shen et al., 2011). The inhibitory deficit is associated with both structural and functional anomalies in the frontostriatal and frontoparietal circuits, highlighting hypoactivation in “go/no go” type tasks compared to the normal population (De La Fuente et al., 2013; Hart et al., 2013). The disorder also consists of considerable difficulties in the modulation of affective states, due to alterations in motor control (Sobanski et al., 2010), and in the recognition and understanding of emotional information and all this often translates into aggression, irritability, or frustration (Martel and Nigg, 2006). For this reason, various pharmacological, cognitive-behavioral, and family treatments are recommended (Taylor et al., 2004). However, cognitive-behavioral intervention strategies have not always proven effective, especially with regard to generalization and long-term effects (Cortese et al., 2015; Evans et al., 2018). Therefore, we tried to find other effective strategies in improving the attention and self-management skills of patients suffering from ADHD.

Research on the rhythms of brain electrical activity in various frequency bands highlights that anatomically complex homeostatic systems regulate the power spectrum of the EEG. Cortical, thalamic, and brainstem processes mediate this regulation through major neurotransmitters, so a deficiency or excess of any neurotransmitter can produce a change in the EEG spectrum and contribute to psychiatric pathophysiology (Coburn et al., 2006). Conditions such as epilepsy, anxiety disorder, depression, dementia, obsessive-compulsive disorder, schizophrenia, learning disability, and ADHD present abnormal patterns in brain electrical activity (Popa et al., 2020). The presence of an excessive amount of slow waves in the frontal areas of the brain leads to difficulty in controlling attention, behavior, and emotions. In this case, people usually present problems with concentration, memory, impulse control, and hyperactivity, along with dysfunctions in focus and intellectual efficiency (Hammond, 2011). The discovery of alterations in the rhythms of brain electrical activity has led to the demonstration that these rhythms play an important role in the maintenance of brain function and, consequently, can be used in the diagnosis of brain dysfunction (Britton et al., 2016). Furthermore, as previously highlighted, the ability to modify specific patterns of brain activity leads to the possibility of using other therapies or alternative treatments, such as neurofeedback (Sitaram et al., 2017).

The main goal of neurofeedback in the treatment of ADHD is to help the brain regulate activity in neural circuits responsible for attention and self-control (Enriquez-Geppert et al., 2019), encouraging a reduction in their activity dysfunctional and promoting the activation of regions related to calm and concentration (Thompson and Thompson, 2023). Over the past 5 years, studies using neurofeedback have reported consistent small to medium sized effects in symptom improvement. In 2019, Enriquez-Geppert and collaborators provided an overview of the use of NF in the treatment of ADHD, examining the scientific evidence on its effectiveness and clinical implementations. Based on meta-analysis studies, three standard NF protocols (theta/beta, sensorimotor rhythm, and slow cortical potential) have been identified that have proven effective in the treatment of ADHD. However, there are currently no uniform treatment standards in the clinical setting. This leads to the need to regulate NF as a therapy and to have application standards at an international level. The same authors noted that thanks to NF a patient can reduce symptoms of impulsivity and improve concentration, successfully interrupting drug therapy (Enriquez-Geppert et al., 2019). However, pharmacological therapy remains a fixed and essential point for treating the symptoms of ADHD. In the same year, other authors conducted a systematic review with the aim of comparing the efficacy and tolerability of methylphenidate (MPH) and NF as treatments for ADHD. The results showed that the two treatments do not show any difference, although this conclusion is not unanimously accepted (Yan et al., 2019). A 2022 study investigated the potential additional effects of NF when combined with drug therapy, finding that NF combined with medications shows positive effects on global ADHD symptomatology and inattention symptoms, even if the effects do not persist for more than 6 months after treatment. Overall, the findings support the idea that supplementing NF with medications provides additional benefits in the treatment of global and inattention symptoms in ADHD patients (Lin et al., 2022).

In 2021, Baena and colleagues investigated the effectiveness of NF interventions in children with ADHD and showed the utility of NF in improving ADHD symptomatology in children through learning appropriate video training (Luo et al., 2023). NF has been shown to have positive and long-lasting effects on ADHD symptoms, significantly improving behavior, attention, IQ and reaction times, with related benefits in motor control and bimanual coordination, which are often problematic in children with ADHD (Sampedro Baena et al., 2021). In a further meta-analysis, some authors examined the efficacy of NF in the treatment of core symptoms of ADHD, highlighting significant improvements in symptoms of inattention and hyperactivity/impulsivity and in global symptoms of ADHD, which appear to be associated with a reduction in theta waves (Fullen et al., 2020). However, according to some authors, NF remains a controversial approach, arguing that it is a purely experimental method that cannot be considered valid for ADHD. In fact, although many studies on NF have reported positive effects, Zilverstand et al. (2017) highlighted conflicting results, finding positive effects only on cognitive functioning. Neurofeedback intervention aimed at reducing the theta/beta ratio represents the main choice for the treatment of ADHD (Van Doren et al., 2017). However, its results have not been fully proven (Neuhäußer et al., 2023). The presence of individual variations in EEG tracings could explain this ineffectiveness (Bluschke et al., 2016). In fact, it has been seen how the alpha rhythm (which plays an important role in the cognitive, psychomotor, psychoemotional, and physiological functions of the brain) could be mistakenly considered as high theta activity according to the normal frequency ranges (Escolano et al., 2014). This has led to evidence that children with ADHD have a higher theta/alpha ratio, in addition to the theta/beta ratio (Janssen et al., 2020). NF targeting theta/beta ratio could negatively affect alpha activity if frequency bands are not identified correctly. Therefore, it is important to track individual frequency bands based on peak frequency and alpha band width. Furthermore, it has emerged that individual variability in the EEG can influence irrelevant areas of the brain if not taken into account (MacDonald et al., 2009) and this could have a negative impact on ADHD-related activities, risking worsening symptoms instead of improving them.

Recent studies have highlighted that, although a significant subgroup оf individuals with ADHD exhibit patterns оf hypoactivation, there іs also a pattern оf hyperactivation іn other cases. This diversity іn activation patterns has been linked tо variability іn responses tо pharmacological treatments, such as the alpha channel blocker guanfacine and the differences іn the effectiveness оf dopamine vs. noradrenaline reuptake inhibitors. Research conducted by Clark et al. (2022), Arns et al. (2020), as well as Kropotov (2020), has demonstrated these differences іn EEG patterns, suggesting that the hypoactivation hypothesis cannot be universally applied tо all individuals with ADHD. This variability underscores the importance оf considering a personalized approach іn the diagnosis and treatment оf ADHD, taking into account the specific neurophysiological characteristics оf the individual. In addition tо the discussion оn the effect оf an excessive amount оf slow waves іn the frontal area compromising control, recent studies, such as those conducted by Clark et al. (2022), have highlighted how an excessive presence оf fast waves, specifically the “beta-spindling” phenomenon, can have a similar impact. This evidence broadens our understanding оf how specific variations іn brain activity patterns can influence cognitive and behavioral control. The elevated presence оf beta activity, associated with states оf anxiety, hyper-vigilance, оr stress, suggests that both slow and fast oscillations must be considered іn assessing brain functionality and designing therapeutic interventions. Therefore, іn our study, we have included a more comprehensive assessment оf brain activity patterns, recognizing that both hypoactivation and hyperactivation can contribute tо behavioral and cognitive dysfunctions іn various clinical conditions.

Some authors suggest that NF causes non-specific effects, improving concentration, self-efficacy and the ability to sit still in children, but these changes may not only be due to cortical regulation, but also to other factors such as breathing or eye movements (Karjalainen et al., 2023). To establish the specific efficacy of cortical regulation in NF it is important to use adequate controls. A common control modality is “sham neurofeedback,” which provides non-specific or non-real signals to a control group in order to determine whether the changes observed in the experimental group are attributable to the effects of real NF (Schönenberg et al., 2017). However, the use of fake NF can lead to methodological difficulties and ethical concerns, as it involves the administration of fake feedback to children with ADHD who in this way, despite having contributed to the experimental project, were not able to derive any benefits from the treatment.

In addition оn the methodological critique оf neurofeedback (NF), іt іs imperative tо consider the analogy between sham controls іn NF research and placebo controls іn medication studies. Recent discussions, as highlighted by studies such as those by Maneeton et al. (2015) have emphasized the substantial effects оf placebos іn medication trials. This іs particularly relevant when comparing the efficacy оf medications like methylphenidate (MPH) against placebo, where the difference, albeit significant, also underscores the non-negligible impact оf placebo itself. Considering this, we propose a nuanced exploration оf the placebo effect іn medication trials relative tо sham NF controls. This involves questioning whether the baseline tо post-treatment placebo effect іs indeed smaller than the interaction effect observed іn comparisons like MPH(t1 − t2) − Placebo(t1 − t2). Such an inquiry іs not only pivotal for understanding the magnitude оf placebo effects but also for appreciating the complexity оf interpreting NF efficacy against these backgrounds. This approach underscores the importance оf rigorous, comparative study designs іn elucidating the true value оf NF іn contrast to, and potentially іn combination with, traditional pharmacological treatments.

We acknowledge that, alongside our discourse оn ADHD and the T/B (Theta/Beta) methodology, іt іs critical tо incorporate an analysis оf Slow Cortical Potentials (SCP, according tо Birbaumer, 1999) and Infra-Low Frequency (ILF). SCPs offer EMG control via the oculogram, essential for ADHD clients during specific parts оf the training that require EEG control, such as during the 8-s phase оf the SCPs, making іt difficult for ADHD clients not tо blink. Studies like those by Strehl et al. (2017) have demonstrated the effectiveness оf EMG as a control group, highlighting that even іn methodologies that include EMG control, the training could be beneficial. Moreover, the integration оf ILF, as discussed іn special editions оf Frontiers, adds an additional layer оf complexity and potential efficacy tо the treatment оf ADHD, offering a deeper understanding and new avenues for therapeutic intervention. Regarding medication, іt іs known that many children dо not tolerate Methylphenidate (MPH) due tо side effects such as premature graying, insomnia, and psychotic symptoms. If Theta/Beta Neurofeedback training proves tо be as effective as MPH without these side effects, patients should be informed оf the existence оf this therapeutic alternative.

An alternative to NF could be the use of electromyographic BF (EMG-BF), which targets motor control rather than regulation of cortical activity. This method could represent a type of control suitable for investigating the specificity of the effects of NF. It was noted that the electromyogenic (EMG) signal generated by the forehead muscles could explain the poor efficacy of NF in ADHD. Elevated forehead muscle tone is considered a sign of psychoemotional tension or mental stress, which may be present in ADHD, and this has led to the hypothesis that the effectiveness of NF treatment may further increase if combined with a practice of EMG-BF (Barth et al., 2017).

In 2013, some authors attempted to demonstrate a differential EMG-BF training method for children with ADHD through the use of different NF training protocols. This method was designed as control training. EMG-BF was used to monitor the activity of arm muscles involved in fine motor skills, such as writing and grip strength control. The authors managed to demonstrate improvements in motor regulation in various task conditions, achieving a significant reduction in behavioral symptoms related to ADHD. The differential EMG-BF approach is found to be useful and provides adequate control conditions for NF training in ADHD research (Maurizio et al., 2013). In 2011, some authors put forward the idea of comparing two types of training to evaluate the effects on the primary symptoms of ADHD: NF to reduce the theta/beta ratio and EMG-BF aimed at relaxing the frontal muscles. For this study, 35 children with ADHD were randomly assigned to the NF group (18 participants) and the BF control group (17 participants). Both groups received 30 treatment sessions. The results showed that the NF group effectively reduced theta/beta ratios and EMG level, and BF achieved a positive impact similar to NF. The study demonstrated that NF is effective in reducing symptoms of inattention in children with ADHD, but suggests that other factors may also influence symptom improvements (Bakhshayesh et al., 2011).

Bazanova et al. (2018) conducted a study to evaluate the effectiveness of a personalized NF intervention based on the theta/beta ratio by combining it with the frontal EMG-BF protocol, in order to treat 94 children with ADHD aged between 6 and 9 years. The experimental design involved randomly dividing participants into four groups in order to reduce the theta/beta ratio (TBR): standard NFT group (sNFT; *n* = 17) with standard frequency bands (4–8 Hz for theta and 12, 5–20 Hz for beta); individual neurofeedback training group (iNFT; *n* = 31), in which theta and beta target frequency bands were individually adapted for each participant; individual NFT group with simultaneous EMG (iNFT_EMG; *n* = 32) where participants received a customized NFT with targeted adjustments to frequency bands and integrated frontalis muscle EMG power to reduce muscle tension; placebo group (sham NF).

The researchers followed a conventional Neurofeedback Training (NFT) protocol to address attention deficit hyperactivity disorder (ADHD) by reducing Theta-beta ratio (TBR) levels, referring to work of Monastra (2005). In this case, the participants underwent 10 sessions of NF each lasting 16 min with their eyes open. During these training sessions, participants sitting in front of a computer monitor and tasked with modulating their TBR were encouraged to develop a mental strategy to reduce their TBR.

The reward criteria were set such that TBR values exceeded a certain threshold in the respective groups. The threshold calculation varied as follows: For the sNFT group, the threshold was based on the average standard frequency power (4–8 Hz for theta and 12–15 Hz for beta). For the iNFT and iNFT_EMG groups, the thresholds were based on the average power of the individually adjusted frequency ranges, while in the iNFT_EMG group the average power of the integrated frontal EMG was considered. In this case the feedback signal appeared only when both the TBR and EMG signals fell below their respective thresholds.

The results of the study confirmed most of the findings on the electrophysiological profile of ADHD, demonstrating a capacity of alpha and beta powers, as well as a leftward shift of the peak frequency of the individual alpha wave in ADHD compared to the control group. Furthermore, it has been shown that personalized NF is more effective in improving attention and impulse control in children with ADHD than the standard protocol and that the effect is more long-lasting when combined with simultaneous EMG control. In conclusion, the effectiveness of NF can be increased by taking into account individual characteristics of EEG and muscle tension (Bazanova et al., 2018).

Research has shown that individuals with ADHD exhibit differences in specific measures of HRV (Shaffer et al., 2014). It has been seen that heart rate variability can provide important information on sustained attention and disturbances in emotional and behavioral regulation observed in ADHD (Griffiths et al., 2017). HRV is a therapeutic approach that aims to train individuals to control a specific aspect of HRV known as respiratory sinus arrhythmia (RSA) (Lehrer and Gevirtz, 2014). The goal of this intervention is to increase heart rate variability leading to positive effects on psychological well-being, such as the reduction of stress and symptoms of anxiety, depression, and post-traumatic stress disorder (PTSD; Gevirtz, 2013). Lloyd et al. (2010) subjected children with ADHD to HRV training and showed that this treatment helps reduce several behavioral symptoms of ADHD, providing a promising non-pharmacological treatment strategy for individuals with this condition.

In conclusion, ADHD is a disorder characterized by symptoms that can affect the daily functioning of individuals who suffer from it. Traditional interventions, such as drug treatment and cognitive-behavioral therapy, can be effective but have limitations, including side effects of medications and uncertain long-term outcomes for behavioral therapies. Despite some methodological controversies, NF represents a promising therapeutic option for the treatment of ADHD, with the possibility of reducing drug dependency and improving patients’ quality of life. The demonstration is given by the fact that Neurofeedback, in particular that aimed at reducing the theta/beta ratio, can have positive effects on the symptoms of ADHD. However, there are individual variations in EEG tracings that can influence the effectiveness of the treatment. For this reason it is important to consider individual factors when designing neurofeedback training. Combining neurofeedback with electromyographic biofeedback (EMG-BF) targeting muscle control may further improve outcomes in the treatment of ADHD. This combination appears to have positive effects on the global symptomatology of ADHD and on the reduction of frontal muscle tension. Despite the positive effects observed, there is a need to regulate neurofeedback as a valid therapy for ADHD and establish application standards internationally to ensure consistent and reliable results.

### 5.4 Autism

Autism Spectrum Disorder (ASD) is an early-onset neurodevelopmental disorder characterized by difficulties in social interaction and communication, restricted interests and repetitive and stereotyped behaviors, and deficits in executive functions and emotional regulation (APA, 2013). The latest revision of the Statistical and Diagnostic Manual of Mental Disorders, the DSM-5 (APA, 2013), has modified the diagnostic classification of this pathology by introducing the concept of “autism spectrum” (which includes four independent diagnoses from the previous DSM-IV: autistic disorder, Asperger syndrome, pervasive developmental disorder not otherwise specified and childhood disintegrative disorder) and moving from a categorical diagnosis typical of previous versions of the manual to a dimensional diagnosis, in which the level of severity of the symptoms, or of general clinical problem, takes on a central role in the diagnostic process. Therefore, each characteristic, symptom or deficit is defined along a continuum within which intensity and severity can be placed. From a neurobiological point of view, ASD is characterized by impairments in some brain regions, such as the amygdala and the facial fusiform area (Adolphs et al., 2001; Schultz et al., 2003), and by alterations in connectivity functional within and between brain networks, such as the Default Mode Network, the Salience Network, the Executive Control Network, and the Mirror Neuron System (Kennedy et al., 2006; Uddin and Menon, 2009; Shih et al., 2010; Fishman et al., 2014). These anomalous connectivity patterns may underlie the disorganized and dysfunctional integration of information that characterizes individuals with ASD (Brock et al., 2002; Belmonte et al., 2004).

In addressing the critical insights into neurofeedback (NF) applications within Autism Spectrum Disorder (ASD), іt іs imperative tо acknowledge the pioneering work оf Robert Coben. His research has significantly contributed tо our understanding оf the neural underpinnings оf ASD, particularly regarding the patterns оf over and underconnectivity observed іn individuals with ASD (Coben et al., 2008, 2010; Coben and Myers, 2008). Coben’s work has elucidated the complex neural connectivity issues that characterize ASD, highlighting the potential for targeted neurofeedback interventions tо ameliorate these specific neural discrepancies. Coben’s innovative approach tо coherence neurofeedback distinguishes his work within the field. Coherence neurofeedback, focusing оn the synchronization between different regions оf the brain, has shown promising results іn improving the symptoms оf ASD, including social interaction, communication, and behavioral flexibility. Coben’s methodology involves the careful assessment and modulation оf brain connectivity, offering a tailored therapeutic intervention that addresses the unique neurophysiological profile оf each individual with ASD.

At a non-central level, however, attention has begun to be paid to the role that the Peripheral Nervous System (PNS) plays in this pathology. According to Polyvagal Theory of Porges (2001, 2003, 2007), the vagus nerve is a mediator of social behavior and, therefore, its dysfunction may contribute to social disorders such as autism. The vagus nerve contributes to the regulation of the Autonomic Nervous System (ANS) through connections with the heart and other visceral organs and is involved in the Social Engagement System (which includes a series of elements related to sociality, such as gaze, facial expression, prosody, etc.), so it is thought that its malfunction could mediate the social avoidance behaviors typical of ASD.

Given the importance attributed to the Central Nervous System (CNS) and the Peripheral Nervous System in the typical dysfunctions of ASD, Goodman et al. (2018) conducted a study aimed at intervening on both top-down (CNS) and bottom-up processes -up (SNP) in order to evaluate its effectiveness in improving ASD symptoms. Specifically, the aim of their study was 2-fold: to evaluate the effect of a BF intervention using HRV (HRV-BF) on ASD symptoms and to examine whether a combination of HRV-BF and NF targeted the Mu rhythm (MRS-NF) was more effective than HRV-BF applied individually. The Mu rhythm was chosen as the target of NF training as it is linked to the activity of the Mirror Neuron System and, therefore, implicated in social and imitation behaviors (Pineda, 2008; Bernier et al., 2013; Braadbaart et al., 2013), compromises in subjects suffering from ASD. Training this brain wave has shown promise in reducing linguistic symptoms and deficits in social cognition and emotional responsivity (Pineda et al., 2008, 2014a; Friedrich et al., 2015). HRV training was instead chosen as it could reflect social behavior based on the principle of neurovisceral integration (Thayer and Lane, 2000). It is not only the CNS that influences the activity of the ANS through efferent connections mediated by the vagus nerve, but also the visceral regions send afferent information to the brain areas. This bidirectional system is known as the Central Autonomic Network (Benarroch, 1993) and some regions of this network (such as the amygdala, the insula and the anterior cingulate and orbitofrontal cortices) overlap with networks involved in attentional, emotional and social processing which play a role in ASD (Sabbagh, 2004; Kana et al., 2007; Di Martino et al., 2009; Uddin and Menon, 2009). This system maintains the homeostatic balance between the CNS and the PNS, therefore HRV training not only acts on the PNS, but also influences the activity of the CNS.

The sample of the study by Goodman et al. (2018) consisted of 15 children diagnosed with ASD divided into two groups: Group 1 underwent HRV-BF training, while Group 2 underwent HRV-BF + MRS-NF training. All children underwent a pre-test phase (T1) consisting of qEEG, Mu rhythm suppression index, baseline HRV, Social Responsiveness Scale-2 (Constantino, 2012), Emotion Regulation Checklist (Shields and Cicchetti, 1997), Spence Children’s Anxiety Scale (Spence, 1998; Nauta et al., 2004), and Autism Treatment Evaluation Checklist (Rimland and Edelson, 1999). They were subsequently subjected to diagnostic tests and four preliminary sessions of HRV-BF. Group 1 was then given an additional 12 h of HRV-BF (in which children were reinforced for breathing at their resonant rate, while punished for breathing faster), while Group 2 was given 12 h of HRV-BF + MRS-NF in which, not only were children reinforced and punished for breathing at their resonant frequency, but they were also reinforced when they increased Mu rhythm levels and punished when they decreased them. Finally, all children underwent a post-test phase similar to the pre-test phase (T2).

The results of this study showed no differences between groups over time in social behavior, autistic symptoms, emotion regulation, anxiety, or HRV. However, Group 1 showed significant improvements in emotional regulation (ŋ^2^ = 0.511) and social behavior (ŋ^2^ = 0.730), while in Group 2 significant improvements occurred in emotional negativity/lability (ŋ^2^ = 0.461), autistic symptoms (ŋ^2^ = 0.499) and in HRV (ŋ^2^ = 0.226). Furthermore, significant time × group differences (ŋ^2^ = 0.364) were found in Mu rhythm suppression according to a pattern contrary to what the authors hypothesized: Group 1 showed a small increase in Mu rhythm suppression, while Group 2 did there is a large reduction in the suppression of this rhythm (i.e., a less adaptive response). In this study, the effect of MRS-NF on Mu rhythm suppression contrasts with what was observed previously in the literature (Pineda, 2005; Pineda et al., 2008, 2014b; Friedrich et al., 2015) and this could be due to several reasons. For example, the ability to suppress the Mu rhythm may require a longer training period than allowed in the study by Goodman and colleagues (Pineda et al., 2008; Friedrich et al., 2015). A second possibility is that synergistic alpha synchronization occurred in the HRV-BF + MRS-NF group, as slow breathing may induce a higher alpha rhythm due to relaxation (Casciaro et al., 2013). Another explanation could be that the training protocol used did not actually provide a reward and, finally, it is possible that the results were distorted by the poorness of the EEG signal or the presence of artifacts during data collection. Regarding HRV-BF, the study by Goodman and colleagues was the first to suggest that this technique can positively influence the symptoms of ASD, as shown by the results obtained from Group 1. However, due to the small number of participants and the lack of a control group, the results obtained by both groups cannot be generalized to the reference population.

Since there are no systematic reviews in the literature relating to the effectiveness of BF training in improving the symptoms of ASD, we will limit ourselves to comparing only the results of MRS-NF training obtained by Goodman et al. (2018) with those reported in the only systematic review to have taken into consideration studies conducted with the NF technique on subjects suffering from ASD, in order to confirm or refute its effectiveness. Kumari and Sharma’s (2020) review includes 17 studies that used NF training to improve social cognition deficits in ASD. However, only five studies used the Mu rhythm-targeted protocol. Of these, the study by Pineda et al. (2014a) showed improvements in social/cognitive awareness and communication, the study by Datko et al. (2018) highlighted positive changes in communication, the studies by Pineda et al. (2008) detected changes in social/cognitive awareness and imitation and the 2015 study by Friedrich and colleagues demonstrated improvements in communication and imitation. Unfortunately, however, due to the heterogeneity of the studies in terms of design, follow-up and presentation of details, Kumari and Sharma were unable to conduct a quantitative pre-post training analysis, so there are no effect size measures that provide robustness to the results obtained, thus not allowing precise conclusions to be drawn. In our literature search we found two other reviews that dealt with the topic of NF in relation to ASD, but they were not included in our work for two reasons. First, in the review by Ribas et al. (2022), the NF interventions were different from the one examined in our review (i.e., the MRS-NFB) and, secondly, although in the work of Hurt et al. (2014) there were four studies based on NF training, only two had used the protocol targeting the Mu rhythm (Pineda et al., 2008) and they were both already mentioned in the 2020 review by Kumari and Sharma cited previously.

In conclusion, by comparing the results obtained by Goodman et al. (2018) with those highlighted by the review by Kumari and Sharma (2020), it is possible to highlight, although not in a rigorous manner, the ability of an MRS-NF intervention to bring about improvements in various aspects of social cognition in individuals with ASD, such as communication, imitation, social/cognitive awareness, and social behavior. However, as previously mentioned, these results cannot be generalized and should be interpreted with caution due to both the small number of studies present in the literature and the methodological limitations they present. Further research characterized by greater methodological rigor (randomization, follow-up, presence of a control group, description of the sample and the procedure and clear explanation of the statistical analysis carried out and the results obtained) are therefore necessary to better understand the potential that a NF intervention has in improving the deficits in social cognition that characterize subjects affected by ASD. Similarly, based on the results obtained by Goodman et al. (2018) and in consideration of the existing interconnection between CNS and ANS, future studies could deepen the use of vagal stimulation training via HRV-BF in order to verify its effectiveness on the social cognition deficits typical of ASD. If the effectiveness of this technique were to be confirmed, it could be used in joint NF + BF experiments, with the aim of establishing its validity and advantages compared to individual approaches applied individually.

## 6 Conclusion and future perspectives

Our discussion synthesizes the new insights garnered from the review, emphasizing the implications for both theory and practice. We delineate the unique contributions оf integrating BF and NF, including the identification оf specific conditions and settings where this approach yields significant benefits. By comparing our findings with existing literature, we spotlight gaps that our review addresses, such as the potential for personalized therapy protocols and the exploration оf combined biofeedback modalities іn diverse populations. Furthermore, we propose a framework for future investigations, underlining the necessity for robust, innovative studies tо fully unravel the complexities оf biological feedback. Ultimately, our review not only sheds light оn previously uncharted territories but also sets the stage for transformative research іn the field.

While іt іs true that the limited number оf studies and the absence оf detailed statistical data, such as effect size measures, necessitate caution іn interpreting the results, this does not entirely preclude the possibility оf drawing meaningful preliminary conclusions. Our review, despite highlighting these limitations, aims tо emphasize the potential оf the integrated BF + NF approach іn improving various conditions and performances, based оn the observed trends іn the available data.

Specifically, we have identified several areas where the integrated approach appears promising, though further research іs needed tо confirm these findings. These areas include improvements іn stress management, attention and impulse control, as well as overall psychological well-being. Additionally, we discussed how treatment personalization based оn specific EEG profiles may represent a significant methodological innovation, enhancing treatment efficacy.

We recognize the importance оf conducting future studies with greater methodological rigor, including active control groups and random assignment оf subjects, tо reduce the risk оf bias and confirm the effectiveness оf the intervention. Furthermore, we emphasize the need for clear and detailed reporting оf statistical analyses and related data, with a particular focus оn effect size measures, tо provide a more robust understanding оf the relationship between combined BF + NF training and the observed outcomes.

The small number of studies collected, together with the lack of statistical data (effect size measures), does not allow us to draw reliable conclusions to support the effectiveness and validity of an integrated BF + NF intervention. Therefore, as mentioned previously, ours represents a description of the literature present to date on the topic, with the aim of highlighting the potential results of using an integrated BF + NF approach and providing a narrative basis for any future studies. Further investigations are in fact necessary to determine the real usefulness of this method. These studies should be characterized by a more rigorous methodology, including an active control group (sham BF + NF) and the randomized assignment of subjects to each group, to reduce the risk of bias and be able to correlate any benefits observed with the treatment being studied of the study, thus limiting the probability of interference by confounding variables. Furthermore, as widely demonstrated by several studies (e.g., Escolano et al., 2014; Bazanova et al., 2018), it would be preferable to use individualized NF protocols, based on quantitative EEG (qEEG) measurements, which take into account consideration of individual variability in EEG profiles (Köpruner et al., 1984; Klimesch, 1999; Clark et al., 2004) and allow the use of personalized protocols for each subject, thus maximizing the probability of treatment success. Furthermore, future research should report clearly and in great detail the type of statistical analysis carried out and the related data obtained, making particular reference to the effect size measures, thanks to which it would be possible to determine the strength of the relationship between the combined BF + NF training and the reduction of the symptoms of a specific pathology or the improvement of sporting or cognitive performance. Finally, follow-ups at multiple time intervals should be foreseen in the experimental procedure, in order to determine whether the results of the combined approach are maintained over time and to quantify their duration.

One final consideration concerns the distinction between the theoretical potential оf integrated biofeedback (BF) and neurofeedback (NF) protocols and their practical application іn clinical settings outside оf academia. In practice, the availability and ease оf use оf combined protocols are largely dependent оn the software and systems provided by vendors. This gap between the theoretical benefits оf integrated BF and NF training and the practical challenges faced by practitioners underscores a critical need. Vendors play a crucial role іn bridging this gap by developing more user-friendly, integrated solutions. Without vendor support іn making these protocols more accessible and easier tо implement, the potential for these innovative treatments to reach patients outside оf academic research remains limited.

In conclusion, while acknowledging the significant contributions оf previous studies, this review has unearthed new insights that enrich our understanding оf the integrated use оf biofeedback and neurofeedback techniques. Specifically, we have identified and highlighted:

- New areas оf application: Our analysis has revealed that, despite the presence оf prior studies, there are specific areas and conditions where the integrated BF + NF approach can be further explored tо maximize therapeutic benefits and performance enhancement.
- Methodological innovations: We have emphasized the importance оf innovative methodological approaches іn the application оf BF and NF techniques, which can offer new perspectives оn their therapeutic potential and mechanisms оf action.
- Practical implications for treatment personalization: Our review suggests that the integration оf BF and NF could be personalized based оn individual needs, offering potential for more targeted and effective treatments.

These insights contribute tо a richer and more nuanced understanding оf the integrated use оf BF and NF, proposing new directions for future research and underscoring the need for further rigorous and methodologically innovative studies. We encourage researchers tо build оn these foundations, exploring uncharted potentials and refining treatment strategies tо further improve clinical and performance outcomes.

## Author contributions

BT: Conceptualization, Investigation, Writing – original draft. SC: Conceptualization, Investigation, Writing – original draft. SM: Methodology, Writing – review & editing. TDL: Methodology, Writing – review & editing. AR: Supervision, Writing – review & editing. AA: Supervision, Writing – review & editing. PD: Supervision, Writing – review & editing.
